# Planning Study of Flattening Filter Free Beams for Volumetric Modulated Arc Therapy in Squamous Cell Carcinoma of the Scalp

**DOI:** 10.1371/journal.pone.0114953

**Published:** 2014-12-15

**Authors:** Youqun Lai, Liwan Shi, Qin Lin, Lirong Fu, Huiming Ha

**Affiliations:** Department of Radiation Oncology, The First Affiliated Hospital of Xiamen University, Xiamen, PR China; University of the Witwatersrand, South Africa

## Abstract

**Purpose:**

Flattening filter free (FFF) beams show the potential for a higher dose rate and lower peripheral dose. We investigated the planning study of FFF beams with their role for volumetric modulated arc therapy (VMAT) in squamous cell carcinoma of the scalp.

**Methods and Materials:**

One patient with squamous cell carcinoma which had involvement of entire scalp was subjected to VMAT using TrueBeam linear accelerator. As it was a rare skin malignancy, CT data of 7 patients with brain tumors were also included in this study, and their entire scalps were outlined as target volumes. Three VMAT plans were employed with RapidArc form: two half-field full-arcs VMAT using 6 MV standard beams (HFF-VMAT-FF), eight half-field quarter-arcs VMAT using 6 MV standard beams (HFQ-VMAT-FF), and HFQ-VMAT using FFF beams (HFQ-VMAT-FFF). Prescribed dose was 25×2 Gy (50 Gy). Plan quality and efficiency were assessed for all plans.

**Results:**

There were no statistically significant differences among the three VMAT plans in target volume coverage, conformity, and homogeneity. For HFQ-VMAT-FF plans, there was a significant decrease by 12.6% in the mean dose to the brain compared with HFF-VMAT-FF. By the use of FFF beams, the mean dose to brain in HFQ-VMAT-FFF plans was further decreased by 7.4% compared with HFQ-VMAT-FF. Beam delivery times were similar for each technique.

**Conclusions:**

The HFQ-VMAT-FF plans showed the superiority in dose distributions compared with HFF-VMAT-FF. HFQ-VMAT-FFF plans might provide further normal tissue sparing, particularly in the brain, showing their potential for radiation therapy in squamous cell carcinoma of the scalp.

## Introduction

Squamous cell carcinoma of the scalp (SCC) is a rare skin malignancy which may be associated with human papillomavirus type 16 [1]. Radiation therapy is an important therapeutic approach [2]–[3]. How to design radiotherapy plan for SCC of the total scalp is an interesting investigative topic. The plan quality is one of the most important pre-treatment prognostic factors in radiation therapy. Whether applying photon-electron fields or intensity modulated radiotherapy (IMRT), neither of them had significant advantage on the dose distributions, for example, low target volume coverage and high hot spot [4].

In recent years, volumetric modulated arc therapy (VMAT) had been widely used in the treatment of various tumors, such as nasopharyngeal carcinoma (NPC), pelvic malignancy, prostate cancer and so on [5]–[7]. Several investigators have studied the role of VMAT for stereotactic body radiotherapy (SBRT) which has been shown to shorten beam on times (BOT) if compared with static gantry IMRT [8]. In addition, Song JH *et al.* [9] have compared lateral photon-electron (LPE), helical tomotherapy (HT), and VMAT plans for total scalp irradiation. High-dose-rate (HDR) brachytherapy has also been used for total scalp irradiation by investigators [10]. Recently, with more and more widespread and profound application of TrueBeam linear accelerator (Varian Medical Systems, Paolo Alto, CA) in radiotherapy, clinical application of flattening filter free (FFF) beams have gradually become the focus of attention [11]–[12]. FFF beams potentially increase dose rates thus reduce treatment delivery time, and lower peripheral dose manifests its unique characteristic. Hrbacek J *et al.* [11] have demonstrated that out-of-field dose deposited by FFF beams is lower than that for flattened beams under most conditions. Georg D *et al.* [13] have also described the lower peripheral dose for FFF beams and the resulting potential advantages in their medical use. At present time, most of the studies focus on the high dose rates of FFF beams for the clinical application to SBRT, such as brain metastases, lung tumors, hepatic metastases and prostate cancer [14]–[17]. In addition, dosimetric comparison was assessed for FFF beams compared with flattened beams for several authors, and the results showed that FFF beams resulted in dose distributions similar to flattened beams [18]–[19].

FFF beams have the potential for a higher dose rate and lower peripheral dose [11]. In this planning study, we assessed that whether these advantages can apply to large elliptical annular field treatment, like the whole scalp radiation therapy, and investigated the planning study of FFF beams for VMAT in squamous cell carcinoma of the scalp.

## Methods and Materials

### Patient selection and contouring

The study was approved by the ethics committee of the First Affiliated Hospital of Xiamen University. All patients provided written consent for storage of their medical information in the hospital database and for research use of this information, and the information of patients was anonymized and de-identified prior to analysis.

One patient with squamous cell carcinoma which had involvement of entire scalp was chosen for analysis in this planning study, and was subjected to VMAT using the TrueBeam linear accelerator. Image guidance was performed by pre treatment cone beam computed tomography (CBCT). As it was a rare skin malignancy, CT data of 7 patients with brain tumors were also included and planed as entire scalp treatment in this study. CT images were acquired by a planning computed tomography scan with 2 mm slice thickness (General Electric Medical Systems, CT Lightspeed 16).

**Figure 1:**
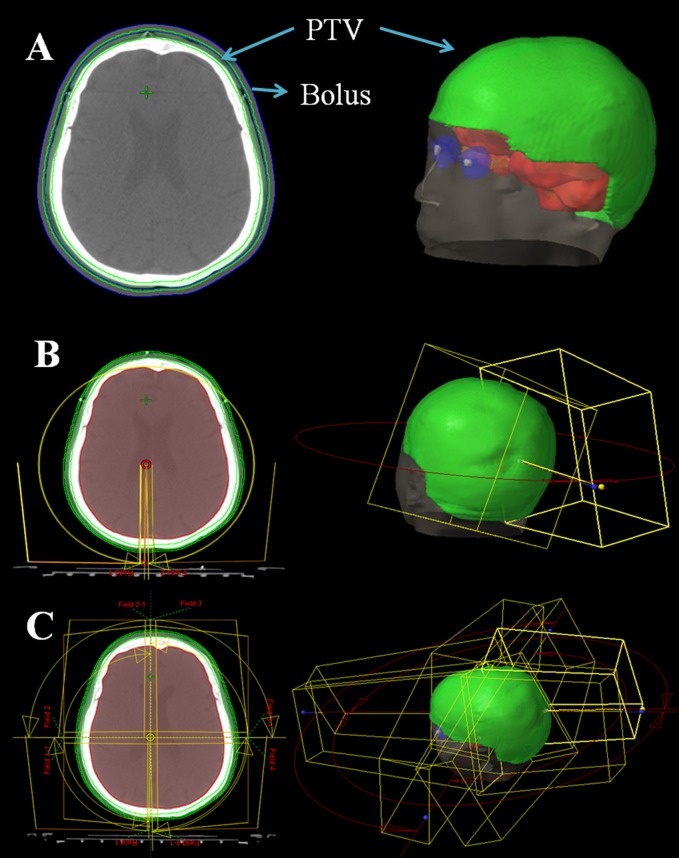
**Delineated planning target volume (PTV) for squamous cell carcinoma of the scalp (A), and two different beam setups in VMAT plans:** (B) Two 360° arcs were used with half-field beam. (C) Eight half-field quarter-arcs (90°) were used in VMAT plan.

Contouring of clinical target volume (CTV) included the entire scalp bordered by the face anteriorly and the neck to the sides and posteriorly. The planning target volume (PTV) was derived from CTV plus a symmetrical 1-mm margin (Fig. 1A). Mean PTV size and standard deviation were 340.8±21.1 cm^3^ (range: 310–357.2 cm^3^). Organs at risk (OAR) such as brain, brain stem, optic nerves, optic chiasma and lens were outlined in the axial CT sections. Mean brain size and standard deviation were 1426.3±50.8 cm^3^ (range: 1355–1448 cm^3^). A 5-mm bolus was applied to the skin surface around PTV to prevent the optimizer compensating for lack of dose in the buildup region during optimization.

### Treatment planning

Treatment planning was performed using two photon beams of TrueBeam SN1402 machine equipped with standard Millennium MLC with 120 leaves. For all patients, plans were designed for three techniques (HFF-VMAT-FF, HFQ-VMAT-FF and HFQ-VMAT-FFF) as described below. 6 MV standard (6X-FF) beams were applied for HFF-VMAT-FF and HFQ-VMAT-FF plans, and 6 MV FFF beams were used for HFQ-VMAT-FFF plans.

All patients were planned to use VMAT (RapidArc, Varian Medical Systems) technique in the Eclipse treatment planning system (Varian Medical Systems, PRO 11.0, AAA 11.0) allowing the optimizer to use the maximum dose rate of 600 MU/min for 6X-FF and 1400 MU/min for 6X-FFF beams. Prescribed dose was 25×2 Gy (50 Gy). For every VMAT plan, the normal tissue objectives and objectives for PTV were kept constant to avoid bias. Doses were normalized so that 95% of PTV received 100% of the prescribed dose and to minimize the volume inside the PTV receiving >110% of the dose. For the OARs, the maximum dose of lens were received <5 Gy, and the mean dose of brain was required to be kept as low as possible.

Actually, the patient with SCC was irradiated with 25×2 Gy (50 Gy) to CTV and 37×2 Gy (74 Gy) to gross tumor volume (GTV) (see S1, S2, and S3 Figure). In this study, for the convenience purpose, only 50 Gy in 2 Gy fractions for the entire scalp was used.

### Two half-field full-arcs VMAT with FF beams (HFF-VMAT-FF)

**Figure 2:**
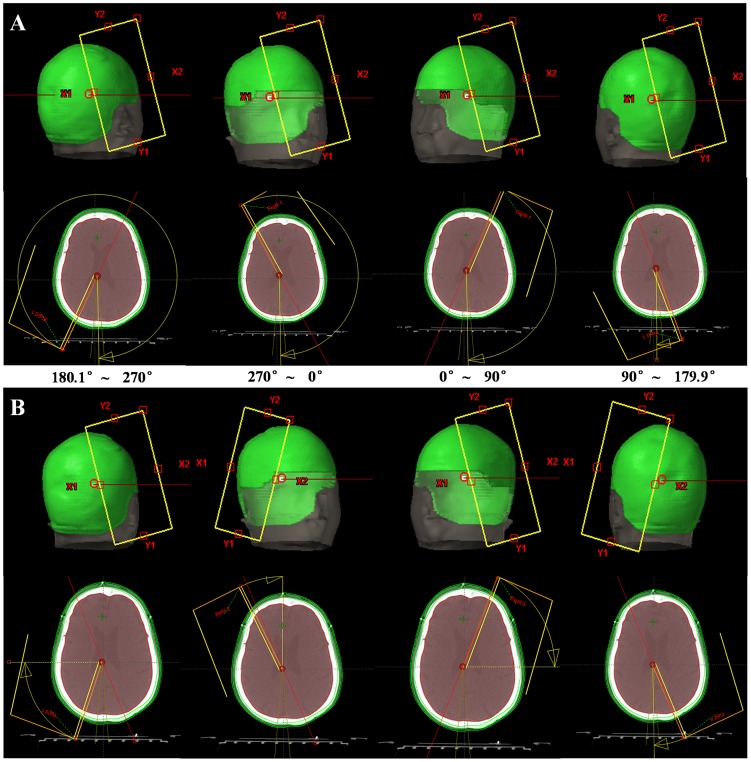
**The beam setup of two designs for VMAT plans in clockwise (CW):** (A) HFF-VMAT: two half-field full-arcs VMAT; (B) HFQ-VMAT: eight half-field quarter-arcs VMAT.

As it was showed in Fig. 1B, two 360° arcs were used with half-field beams. Fig. 2A showed the beam setup of HFF-VMAT plans using 6 MV standard beams. In clockwise (CW) direction, collimator angle was 15° and field was opened at X2 of collimator, and kept invariant from 180.1° to 179.9°. Similarly, in counter-clockwise (CCW) direction, collimator angle was 345° and field was opened at X1 of collimator.

### Eight half-field quarter-arcs VMAT with FF beams (HFQ-VMAT-FF)

For HFQ-VMAT-FF plans, eight half-field quarter-arcs were employed with 6 MV standard beams (Fig. 1C). One 360° arc was divided into four equal sections, since each section was 90°. Fig. 2B showed the beam setup of HFQ-VMAT plans. In CW, collimator angle was 15° and field was opened at X2 of collimator from 180.1° to 270° and 0° to 90°, while collimator angle was 345° and field was opened at X1 of collimator from 270° to 0° and 90° to 179.9°. It was similar in CCW.

### Eight half-field quarter-arcs VMAT with FFF beams (HFQ-VMAT-FFF)

For HFQ-VMAT-FFF plans, 6X-FFF beams (un-flattened beams) were used with the maximum dose rate of 1400 MU/min.

### Plan evaluation and statistical methods

Plan quantitative evaluation was performed according to the standard dose volume histograms (DVH). For the PTV, the values of D_98%_ and D_2%_ (dose received by 98%, and 2% of the target volume) were defined as metrics for minimum and maximum doses. The conformity index (CI) was defined as: CI = (V_PTV_/TV_PV_)/(TV_PV_/V_TV_). V_PTV_ is the volume of target volume. TV_PV_ is the portion of the V_PTV_ within the prescribed isodose line. V_TV_ is the volume of the body that received 100% of the PD. The homogeneity index (HI) was defined as: HI = D_5%_/D_95%_ (dose received by 5%, and 95% of the target volume) [20]. For OARs, we focused on the evaluation of the mean dose for the brain. The mean doses to brain stem, optic nerves, optic chiasma and lens were also reported. Total MUs, beam on time (BOT) and mean dose rate (MU/min) were compared for the three plans.

Statistical analyses were performed by comparing the different techniques using 6X-FF or 6X-FFF beams. Mean values and standard deviation were collected. All data points were normally distributed by one-sample kolmogorov-smirnov test. Relative dosimetric changes were compared using a paired t test: HFF-VMAT-FF vs HFQ-VMAT-FF, and HFQ-VMAT-FF vs HFQ-VMAT-FFF. P≤0.05 was considered statistically significant.

### Dosimetric validation of VMAT_FFF

VMAT treatment plan was delivered to a diode array phantom (ARCCHECK, Sun Nuclear Corp., Melbourne, FL). The ArcCheck was analyzed with the global γ-index method with 3%/3 mm.

## Results

### PTV coverage and dose distribution

**Figure 3:**
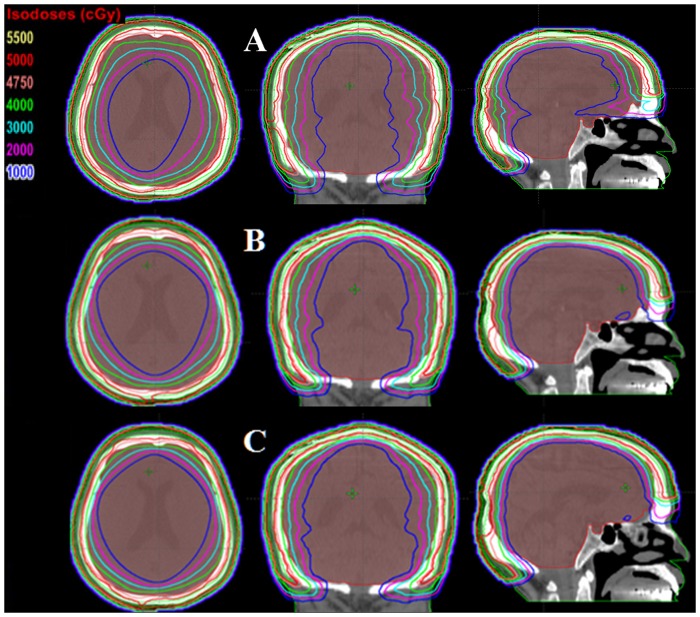
Isodose distributions for one patient with squamous cell carcinoma in axial, coronal, and sagittal planes. (A) HFF-VMAT-FF: two half-field full-arcs VMAT with conventional flattened (FF) beam; (B) HFQ-VMAT-FF: eight half-field quarter-arcs VMAT with FF beam; (C) HFQ-VMAT-FFF: eight half-field quarter-arcs VMAT with flattening filter free (FFF) beam.

**Figure 4:**
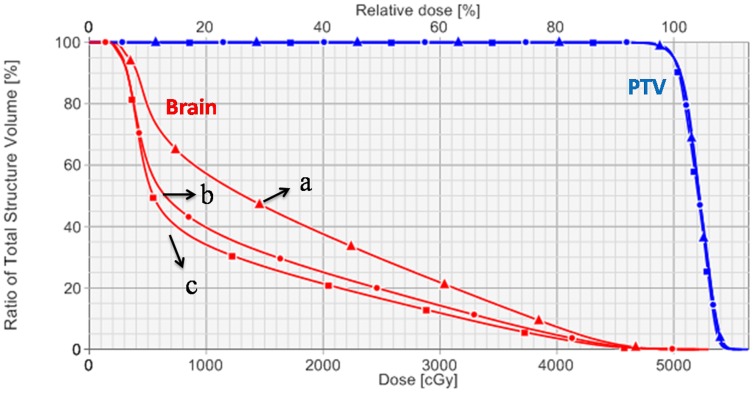
**Dose-volume histogram (DVH) comparison for the PTV and brain with different planning techniques:** (a) HFF-VMAT-FF: two half-field full-arcs VMAT with conventional flattened (FF) beam; (b) HFQ-VMAT-FF: eight half-field quarter-arcs VMAT with FF beam; (c) HFQ-VMAT-FFF: eight half-field quarter-arcs VMAT with flattening filter free (FFF) beam.

As presented in Table 1, dosimetric parameters of PTV for all three groups of treatment plans created with different planning techniques. There were no statistically significant differences (*P*>0.5) among the three VMAT plans in target volume coverage, conformity index (CI), and homogeneity index (HI). The dose distributions were displayed in Fig. 3 for one patient with squamous cell carcinoma in axial, coronal, and sagittal views. Fig. 4 showed dose-volume histogram (DVH) comparison for the PTV and brain with three different planning techniques: (a) HFF-VMAT-FF, (b) HFQ-VMAT-FF, and (c) HFQ-VMAT-FFF.

**Table 1:** Dosimetric parameters of PTV for treatment plans created with different planning techniques.

PTV	HFF-VMAT-FF	HFQ-VMAT-FF	HFQ-VMAT-FFF	*P* value*
D_max_ (Gy)	55.64±0.63	55.57±0.55	55.61±0.59	a = 0.73, b = 0.83
D_min_ (Gy)	41.69±0.45	41.75±0.87	41.71±0.79	a = 0.88, b = 0.9
D_mean_ (Gy)	51.83±0.29	51.86±0.35	51.79±0.25	a = 0.85, b = 0.58
D_2%_ (Gy)	53.60±0.68	53.63±0.74	53.62±0.50	a = 0.88, b = 0.99
D_98%_ (Gy)	49.33±0.15	49.15±0.23	49.32±0.21	a = 0.11, b = 0.23
V_95%_ (%)	99.82±0.07	99.84±0.08	99.88±0.05	a = 0.67, b = 0.31
V_110%_ (%)	0.0067±0.008	0.0037±0.005	0.004±0.005	a = 0.28, b = 0.73
CI	1.40±0.15	1.41±0.14	1.42±0.12	a = 0.35, b = 0.95
HI	1.066±0.012	1.067±0.015	1.066±0.010	a = 0.41, b = 0.47

*Abbreviations:* HFF-VMAT-FF = two half-field full-arcs VMAT with conventional flattened (FF) beam; HFQ-VMAT-FF = eight half-field quarter-arcs VMAT with FF beam; HFQ-VMAT-FFF = eight half-field quarter-arcs VMAT with flattening filter free (FFF) beam; CI = conformity index; HI = homogeneity index.

* *P* value corresponds to the paired *t* test: a = HFF-VMAT-FF vs HFQ-VMAT-FF, b = HFQ-VMAT-FF vs HFQ-VMAT-FFF.

### Dose to organs at risk

Table 2 presented the mean doses of all remaining organs at risk for treatment plans created with different planning techniques. By the use of 6 MV standard beams, in HFQ-VMAT-FF plan, there was a significant decrease by 12.6% (*P*<0.01) in the mean dose to the brain compared with HFF-VMAT-FF plan. When 6 MV FFF beams were used, compared with HFQ-VMAT-FF plans, mean dose to the brain was further decreased by 7.4% (*P*<0.01) for HFQ-VMAT-FFF. Other organs at risk, such as brain stem, optic nerves, optic chiasma and lens, were received low dose level in three VMAT plans.

**Table 2:** The mean doses of organs at risk, MU, beam-on time, and mean dose rate (MDR) for treatment plans created with different planning techniques.

Mean dose	HFF-VMAT-FF	HFQ-VMAT-FF	HFQ-VMAT-FFF	*P* value*
Brain (Gy)	14.61±3.67	12.76±2.66	11.82±2.99	a = 0.01, b = 0.001
Brain stem (Gy)	2.53±0.32	2.11±0.11	2.31±0.24	
lens (Gy)	3.54±0.84	3.04±0.27	2.88±0.43	
optic nerves (Gy)	5.68±2.52	4.31±1.35	4.43±1.36	
optic chiasma (Gy)	4.26±0.48	3.19±0.41	3.44±0.47	
Body (Gy)*	19.9±1.99	18.59±1.49	17.98±1.74	a = 0.013, b = 0.001
MU	922±88	1019±79	1346±130	a = 0.01, b = 0
Beam-on time (s)	241	243	240	
MDR (MU/min)	459±39	502±33	673±65	

*Abbreviations:* HFF-VMAT-FF = two half-field full-arcs VMAT with conventional flattened (FF) beam; HFQ-VMAT-FF = eight half-field quarter-arcs VMAT with FF beam; HFQ-VMAT-FFF = eight half-field quarter-arcs VMAT with flattening filter free (FFF) beam.

* Body: it is the region of the CT which was scanned.

* *P* value corresponds to the paired *t* test: a = HFF-VMAT-FF vs HFQ-VMAT-FF, b = HFQ-VMAT-FF vs HFQ-VMAT-FFF.

### MU and beam delivery time

In total MUs, HFQ-VMAT-FF plans were increased by an average of 10.6% compared with HFF-VMAT-FF, while HFQ-VMAT-FFF plans resulted in an average of 32.1% increase compared to HFQ-VMAT-FF (Table 2). However, though the total MUs were increased, the mean body doses for HFQ-VMAT-FF and HFQ-VMAT-FFF plans were 18.59 Gy and 17.98 Gy, respectively, representing an average 6.9% and 9.6% reduction compared with HFF-VMAT-FF plans. Beam-on times (BOT) were similar for each technique. For the average dose rate, HFF-VMAT-FF, HFQ-VMAT-FF and HFQ-VMAT-FFF plans were 459±39 MU/min, 502±33 MU/min and 673±65 MU/min, respectively.

### Dosimetric validation of VMAT_FFF

In FFF beams, VMAT treatment plan was delivered to ArcCheck. The γ-index analysis of ArcCheck resulted in an excellent agreement of 97±1.5% for 3%/3 mm.

## Discussion

In our study, we designed three VMAT techniques (HFF-VMAT-FF, HFQ-VMAT-FF, and HFQ-VMAT-FFF) and assessed plan quality and efficiency for squamous cell carcinoma of the scalp.

Firstly, we made comparisons between HFF-VMAT and HFQ-VMAT plans with 6X-FF beams. It was evident that the dose distributions in HFQ-VMAT-FF plans were superior to HFF-VMAT-FF (Fig. 3A and Fig. 3B). However, what are the causes of more advantage to HFQ-VMAT plans compared with HFF-VMAT using same energy beams (6X-FF beams)? This can be attributed to the differences in the beam setup between HFF-VMAT and HFQ-VMAT plans. As it was showed in Fig. 2, the difference between two VMAT plans was that collimator angle and field were changed in HFQ-VMAT plans from 270° to 0° and 90° to 179.9°. That is, in CW, collimator angle was always 15° and field was always opened at X2 of collimator for HFF-VMAT plans, while collimator angle was 345° and field was opened at X1 of collimator from 270° to 0° and 90° to 179.9° for HFQ-VMAT plans. Because of these changes, from 270° to 0° and 90° to 179.9° in CW, HFQ-VMAT plans may improve efficiency in PTV coverage and brain sparing due to its elliptical annular target volume of squamous cell carcinoma of the scalp; While in HFF-VMAT plans, it would irradiate more healthy tissue (such as brain) in order to irradiate target volume than in HFQ-VMAT plans. In addition, these changes of HFQ-VMAT plans would be useful only to special elliptical annular target volume.

Secondly, we assessed the role of FFF beams on dosimetry to compare with flattened beams using same beam setup for VMAT plans (HFQ-VMAT). FFF beams show the potential for a higher dose rate and lower peripheral dose, which manifests its unique characteristic. If FFF beams were applied for HFQ-VMAT plans, the mean dose to the brain was further decreased by 7.4% compared with HFQ-VMAT-FF plans (Fig. 3), due to the non-flat profile and the lower peripheral dose for the FFF beam. Zwahlen *et al.* [18] had presented that the integral dose of open fields in a water phantom was smaller than for a flattened beam in relation to 100 MU, and this finding was more pronounced with increasing field size. In HFQ-VMAT plans, a half-field beam was used and field size was about 10∼12 cm due to the large elliptical annular target volume. Under these conditions, the integral dose of peripheral field for FFF beam is decreased by >10% compared with FF beam, and it means that energy is decline in peripheral field. The optimization of VMAT plan for large elliptical annular target volume was to apply peripheral field to irradiate target volume thus to reduce the dose of healthy tissue. Therefore, by the use of FFF beams, the healthy tissue, such as the brain, may be received lower dose than FF beams. In addition, in the case of the lower-energy x-rays, the dose builds up to a maximum on or very close to the surface. But for higher-energy beams, the point of maximum dose lies deeper into the tissue or phantom.

Though the maximum dose rate could reach 1400 MU/min, the average dose rate in HFQ-VMAT-FFF plans was only 673 MU/min (Table 2). For each VMAT technique, BOT was similar due to the VMAT BOT was governed mostly by the gantry speed. Hence, in conventional radiotherapy, about 2 Gy/fraction, the potential for a higher dose rate of FFF beams could not be exploited in VMAT plans. As it was showed in Table 2, a higher number of MU for FFF beams compared with FF beams did not necessarily lead to an increased mean body dose, due to the special non-flat profile of the FFF beam [18].

In summary, we demonstrated that the HFQ-VMAT plans provided more advantages in dose distributions compared with HFF-VMAT plans. The potential for lower peripheral dose of FFF beams would be adequately developed for radiation therapy in squamous cell carcinoma of the scalp.

## Conclusions

The data presented in this report demonstrate that with respect to large elliptical annular target volume, HFQ-VMAT plans with FFF beams showed more advantage on dose distributions, which may have target volume higher dose level while in normal tissues, especially the brain, received minimum dose level. We show that target volume coverage, beam delivery times, CI and HI were similar for each technique. By the use of FFF beams, a higher number of MU compared with FF beams did not necessarily lead to an increased mean body dose. In general, it is feasible to apply FFF beams for VMAT in squamous cell carcinoma of the scalp in clinic.

## Supporting Information

S1 File **The data (file data_PONE-D-14-34378R1.docx) underlying the findings in present study.**
(DOCX)

S2 File **S1 Figure.** The PCTV and PGTV for the patient with SCC. **S2 Figure.** The beam setup and isodose distributions for treatment plan of second phase. Prescribed dose was 12×2 Gy (24 Gy) to PGTV. For the treatment plan of second phase, the patient with SCC was irradiated with 12×2 Gy (24 Gy) to GTV to boost the GTV up to 74 Gy. Four double partial arcs with a maximum individual length of 60° were adopted in this plan. **S3 Figure.** The isodose distributions and hose-volume histogram (DVH) for the sum plan with 25×2 Gy (50 Gy) to PCTV and 37×2 Gy (74 Gy) to PGTV.
(DOCX)
