# Supplementary material for: Planning Study of Flattening Filter Free Beams for Volumetric Modulated Arc Therapy in Squamous Cell Carcinoma of the Scalp
Source: PLoS One. 2014 Dec 15;9(12):e114953. doi: 10.1371/journal.pone.0114953 (PMC4266613; doi:10.1371/journal.pone.0114953)
Supplement: S1 File — The data (file data_PONE-D-14-34378R1.docx) underlying the findings in present study. (DOCX) [file pone.0114953.s001.docx]

| Patients | V_PTV |  |  | TVpv |  |  |
| --- | --- | --- | --- | --- | --- | --- |
|  |  |  |  |  |  |  |
|  | plan1 | plan2 | plan3 | plan1 | plan2 | plan3 |
| 1 | 357.2 | 357.2 | 357.2 | 339.34 | 339.34 | 339.34 |
| 2 | 356.8 | 356.8 | 356.8 | 338.96 | 338.96 | 338.96 |
| 3 | 355.9 | 355.9 | 355.9 | 338.105 | 333.45 | 327.75 |
| 4 | 310 | 310 | 310 | 294.5 | 294.5 | 294.5 |
| 5 | 316.2 | 316.2 | 316.2 | 300.39 | 300.39 | 300.39 |
| 6 | 357 | 357 | 357 | 339.15 | 339.15 | 339.15 |
| 7 | 320.5 | 320.5 | 320.5 | 304.475 | 304.475 | 304.475 |
| 8 | 352.6 | 352.6 | 352.6 | 334.97 | 334.97 | 334.97 |
| Mean | 340.775 | 340.775 | 340.775 | 323.7363 | 323.1544 | 322.4419 |
| SD | 21.11376 | 21.11376 | 21.11376 | 20.05807 | 19.64496 | 19.31886 |
| *P* |  |  |  |  |  |  |

|  | Vtv |  |  | CI |  |  |
| --- | --- | --- | --- | --- | --- | --- |
|  |  |  |  |  |  |  |
|  | plan1 | plan2 | plan3 | plan1 | plan2 | plan3 |
| 1 | 462 | 455.5 | 437.8 | 1.433123 | 1.41296 | 1.358054 |
| 2 | 502 | 507 | 471 | 1.558948 | 1.574475 | 1.462678 |
| 3 | 421 | 405 | 401 | 1.310711 | 1.296348 | 1.328578 |
| 4 | 351 | 355 | 370 | 1.25458 | 1.268877 | 1.322491 |
| 5 | 378 | 381 | 402 | 1.324594 | 1.335106 | 1.408695 |
| 6 | 511 | 498 | 509 | 1.586008 | 1.54566 | 1.579801 |
| 7 | 352 | 371 | 369 | 1.216935 | 1.282622 | 1.275708 |
| 8 | 490 | 510 | 507 | 1.539808 | 1.602657 | 1.59323 |
| Mean | 433.375 | 435.3125 | 433.35 | 1.403088 | 1.414838 | 1.416154 |
| SD | 66.96254 | 64.92795 | 57.03139 | 0.145773 | 0.139929 | 0.119463 |
| *P* |  |  |  |  | 0.347 | 0.947 |
|  |  |  |  |  |  |  |

|  | D5 | |  | |  | | D95 |  |  |
| --- | --- | --- | --- | --- | --- | --- | --- | --- | --- |
|  |  | |  | |  | |  |  |  |
|  | plan1 | | plan2 | | plan3 | | plan1 | plan2 | plan3 |
| 1 | 5395 | | 5402 | | 5368 | | 5000 | 5000 | 5000 |
| 2 | 5346 | | 5390 | | 5357 | | 5000 | 5000 | 5000 |
| 3 | 5265 | | 5246 | | 5274 | | 5000 | 5000 | 5000 |
| 4 | 5301 | | 5311 | | 5305 | | 5000 | 5000 | 5000 |
| 5 | 5405 | | 5411 | | 5393 | | 5000 | 5000 | 5000 |
| 6 | 5281 | | 5272 | | 5265 | | 5000 | 5000 | 5000 |
| 7 | 5385 | | 5407 | | 5394 | | 5000 | 5000 | 5000 |
| 8 | 5271 | | 5255 | | 5289 | | 5000 | 5000 | 5000 |
| Mean | 5331.125 | | 5336.75 | | 5330.625 | | 5000 | 5000 | 5000 |
| SD | 58.63796 | | 73.01223 | | 53.32096 | | 0 | 0 | 0 |
| *P* |  | |  | |  | |  |  |  |
|  | HI |  | |  | |  |  |  |  |
|  |  |  | |  | |  |  |  |  |
|  | plan1 | plan2 | | plan3 | |  |  |  |  |
| 1 | 1.079 | 1.0804 | | 1.0736 | |  |  |  |  |
| 2 | 1.0692 | 1.078 | | 1.0714 | |  |  |  |  |
| 3 | 1.053 | 1.0492 | | 1.0548 | |  |  |  |  |
| 4 | 1.0602 | 1.0622 | | 1.061 | |  |  |  |  |
| 5 | 1.081 | 1.0822 | | 1.0786 | |  |  |  |  |
| 6 | 1.0562 | 1.0544 | | 1.053 | |  |  |  |  |
| 7 | 1.077 | 1.0814 | | 1.0788 | |  |  |  |  |
| 8 | 1.0542 | 1.051 | | 1.0578 | |  |  |  |  |
| Mean | 1.066225 | 1.06735 | | 1.066125 | |  |  |  |  |
| SD | 0.011728 | 0.014602 | | 0.010664 | |  |  |  |  |
| *P* |  | 0.413 | | 0.469 | |  |  |  |  |

|  | Dmax |  |  | Dmin |  |  |
| --- | --- | --- | --- | --- | --- | --- |
|  | plan1 | plan2 | plan3 | plan1 | plan2 | plan3 |
| 1 | 5641 | 5591 | 5566 | 4207 | 4186 | 4235 |
| 2 | 5590 | 5587 | 5628 | 4181 | 4040 | 4053 |
| 3 | 5466 | 5493 | 5489 | 4120 | 4300 | 4226 |
| 4 | 5630 | 5590 | 5568 | 4219 | 4211 | 4171 |
| 5 | 5552 | 5450 | 5495 | 4169 | 4146 | 4231 |
| 6 | 5570 | 5563 | 5625 | 4218 | 4223 | 4241 |
| 7 | 5584 | 5595 | 5501 | 4100 | 4230 | 4055 |
| 8 | 5480 | 5583 | 5612 | 4140 | 4065 | 4156 |
| Mean | 5564.125 | 5556.5 | 5560.5 | 4169.25 | 4175.125 | 4171 |
| SD | 63.47201 | 54.56844 | 59.03752 | 45.49961 | 87.45356 | 78.58208 |
| *P* |  | 0.73 | 0.83 |  | 0.88 | 0.9 |

|  | Dmean |  |  | D2 |  |  |
| --- | --- | --- | --- | --- | --- | --- |
|  | plan1 | plan2 | plan3 | plan1 | plan2 | plan3 |
| 1 | 5213 | 5210 | 5198 | 5429 | 5413 | 5395 |
| 2 | 5182 | 5204 | 5190 | 5361 | 5400 | 5388 |
| 3 | 5155 | 5146 | 5151 | 5292 | 5278 | 5304 |
| 4 | 5218 | 5215 | 5180 | 5425 | 5421 | 5406 |
| 5 | 5162 | 5156 | 5155 | 5357 | 5415 | 5309 |
| 6 | 5138 | 5215 | 5215 | 5289 | 5285 | 5393 |
| 7 | 5203 | 5132 | 5195 | 5438 | 5261 | 5409 |
| 8 | 5195 | 5211 | 5145 | 5270 | 5431 | 5296 |
| Mean | 5183.25 | 5186.125 | 5178.625 | 5357.625 | 5363 | 5362.5 |
| SD | 29.07994 | 35.09757 | 25.49475 | 68.45215 | 73.94399 | 49.85407 |
| *P* |  | 0.85 | 0.58 |  | 0.88 | 0.99 |

|  | D98 |  |  | V95 |  |  |
| --- | --- | --- | --- | --- | --- | --- |
|  | plan1 | plan2 | plan3 | plan1 | plan2 | plan3 |
| 1 | 4923 | 4903 | 4944 | 99.82 | 99.75 | 99.91 |
| 2 | 4926 | 4901 | 4908 | 99.89 | 99.86 | 99.94 |
| 3 | 4950 | 4942 | 4945 | 99.75 | 99.92 | 99.87 |
| 4 | 4928 | 4929 | 4948 | 99.9 | 99.91 | 99.82 |
| 5 | 4916 | 4951 | 4903 | 99.69 | 99.78 | 99.93 |
| 6 | 4956 | 4915 | 4911 | 99.83 | 99.93 | 99.81 |
| 7 | 4937 | 4886 | 4952 | 99.87 | 99.81 | 99.86 |
| 8 | 4928 | 4895 | 4945 | 99.81 | 99.72 | 99.92 |
| Mean | 4933 | 4915.25 | 4932 | 99.82 | 99.835 | 99.8825 |
| SD | 13.74253 | 23.30389 | 20.68816 | 0.071514 | 0.081591 | 0.050071 |
| *P* |  | 0.11 | 0.23 |  | 0.67 | 0.31 |

|  | V110 |  |  |
| --- | --- | --- | --- |
|  | plan1 | plan2 | plan3 |
| 1 | 0.019 | 0.01 | 0.0024 |
| 2 | 0.001 | 0.001 | 0.01 |
| 3 | 0 | 0 | 0 |
| 4 | 0.0016 | 0.0002 | 0.0025 |
| 5 | 0.02 | 0.001 | 0.008 |
| 6 | 0.004 | 0.007 | 0.001 |
| 7 | 0 | 0 | 0 |
| 8 | 0.008 | 0.01 | 0.011 |
| Mean | 0.0067 | 0.00365 | 0.004363 |
| SD | 0.008328 | 0.004543 | 0.004563 |
| *P* |  | 0.28 | 0.73 |

|  | brain |  |  | brain-stem | |  |
| --- | --- | --- | --- | --- | --- | --- |
| mean | plan1 | plan2 | plan3 | plan1 | plan2 | plan3 |
| 1 | 1738 | 1307 | 1176 | 265 | 211 | 225 |
| 2 | 1602 | 1527 | 1484 | 277 | 221 | 257 |
| 3 | 1044 | 996 | 872 | 217 | 200 | 210 |
| 4 | 1650 | 1338 | 1298 | 266 | 215 | 259 |
| 5 | 1023 | 950 | 776 | 284 | 219 | 221 |
| 6 | 1798 | 1533 | 1479 | 217 | 209 | 201 |
| 7 | 1014 | 982 | 889 | 215 | 191 | 215 |
| 8 | 1820 | 1578 | 1475 | 285 | 225 | 258 |
| Mean | 1461.125 | 1276.375 | 1181.125 | 253.25 | 211.375 | 230.75 |
| SD | 366.5131 | 266.2356 | 298.9345 | 31.41769 | 11.33815 | 23.66885 |
| *P* |  |  |  |  |  |  |

|  | lens |  |  | optic-nerve | |  |
| --- | --- | --- | --- | --- | --- | --- |
| mean | plan1 | plan2 | plan3 | plan1 | plan2 | plan3 |
| 1 | 451 | 331 | 330 | 857 | 587 | 599 |
| 2 | 311 | 306 | 301 | 453 | 340 | 349 |
| 3 | 300 | 276 | 245 | 394 | 368 | 380 |
| 4 | 458 | 332 | 329 | 878 | 590 | 601 |
| 5 | 289 | 278 | 231 | 382 | 328 | 335 |
| 6 | 283 | 280 | 275 | 372 | 342 | 368 |
| 7 | 455 | 340 | 338 | 333 | 295 | 299 |
| 8 | 288 | 286 | 251 | 875 | 601 | 615 |
| Mean | 354.375 | 303.625 | 287.5 | 568 | 431.375 | 443.25 |
| SD | 83.50524 | 27.1816 | 42.67485 | 252.3059 | 135.1147 | 136.1268 |
| *P* |  |  |  |  |  |  |

|  | optic chiasma | |  |
| --- | --- | --- | --- |
| mean | plan1 | plan2 | plan3 |
| 1 | 460 | 288 | 294 |
| 2 | 448 | 303 | 349 |
| 3 | 370 | 366 | 389 |
| 4 | 465 | 279 | 299 |
| 5 | 456 | 278 | 305 |
| 6 | 365 | 366 | 405 |
| 7 | 475 | 298 | 312 |
| 8 | 371 | 370 | 395 |
| Mean | 426.25 | 318.5 | 343.5 |
| SD | 48.31962 | 41.32796 | 46.96503 |
| *P* |  |  |  |

|  | Dmean-body | |  | MU |  |  |
| --- | --- | --- | --- | --- | --- | --- |
|  | plan1 | plan2 | plan3 | plan1 | plan2 | plan3 |
| 1 | 2151 | 1866 | 1782 | 859 | 1051 | 1369 |
| 2 | 2052 | 2004 | 1980 | 883 | 929 | 1206 |
| 3 | 1767 | 1706 | 1633 | 1023 | 1078 | 1465 |
| 4 | 1851 | 1726 | 1658 | 866 | 1097 | 1452 |
| 5 | 2198 | 1879 | 1752 | 1048 | 1102 | 1520 |
| 6 | 1701 | 1675 | 1601 | 850 | 986 | 1235 |
| 7 | 2214 | 2092 | 2080 | 1006 | 1025 | 1351 |
| 8 | 1978 | 1920 | 1901 | 840 | 887 | 1170 |
| mean | 1989 | 1858.5 | 1798.375 | 921.875 | 1019.375 | 1346 |
| sd | 198.5346 | 148.5338 | 173.7247 | 87.55967 | 79.39582 | 130.452 |
| *P* |  | 0.013 | 0.003 |  | 0.01 | 0 |
|  |  |  |  |  |  |  |

|  | （BOT) |  |  | mean dose rate | |  |
| --- | --- | --- | --- | --- | --- | --- |
|  | plan1 | plan2 | plan3 | plan1 | plan2 | plan3 |
| 1 | 2 | 2.03 | 2 | 429.5 | 517.734 | 684.5 |
| 2 | 1.99 | 2 | 2 | 443.7186 | 464.5 | 603 |
| 3 | 2.03 | 2.05 | 2 | 503.9409 | 525.8537 | 732.5 |
| 4 | 2.01 | 2.05 | 2.01 | 430.8458 | 535.122 | 722.3881 |
| 5 | 1.98 | 2 | 2 | 529.2929 | 551 | 760 |
| 6 | 2.05 | 2.08 | 1.99 | 414.6341 | 474.0385 | 620.603 |
| 7 | 2 | 2.04 | 2.02 | 503 | 502.451 | 668.8119 |
| 8 | 2.02 | 2.05 | 2 | 415.8416 | 432.6829 | 585 |
| mean | 2.01 | 2.0375 | 2.0025 | 458.8467 | 502.6959 | 673.3333 |
| sd | 0.022678 | 0.027124 | 0.008864 | 39.51864 | 33.32681 | 65.46819 |
| *P* |  |  |  |  |  |  |
