# Supplementary material for: Planning Study of Flattening Filter Free Beams for Volumetric Modulated Arc Therapy in Squamous Cell Carcinoma of the Scalp
Source: PLoS One. 2014 Dec 15;9(12):e114953. doi: 10.1371/journal.pone.0114953 (PMC4266613; doi:10.1371/journal.pone.0114953)
Supplement: S2 File — S1 Figure. The PCTV and PGTV for the patient with SCC. S2 Figure. The beam setup and isodose distributions for treatment plan of second phase. Prescribed dose was 12×2 Gy (24 Gy) to PGTV. For the treatment plan of second phase, the patient with SCC was irradiated with 12×2 Gy (24 Gy) to GTV to boost the GTV up to 74 Gy. Four double partial arcs with a maximum individual length of 60° were adopted in this plan. S3 Figure. The isodose distributions and hose-volume histogram (DVH) for the sum plan with 25×2 Gy (50 Gy) to PCTV and 37×2 Gy (74 Gy) to PGTV. (DOCX) [file pone.0114953.s002.docx]

**Supporting Information for**

Planning Study of Flattening Filter Free Beams for Volumetric Modulated Arc Therapy in Squamous Cell Carcinoma of the Scalp

Youqun Lai, M.S., Liwan Shi, B.Sc., Qin Lin, M.D., Ph.D., Lirong Fu, B.Sc., Huiming Ha, B.Sc.

*Department of Radiation Oncology, The First Affiliated Hospital of Xiamen University, 55, Zhenhai Road, Xiamen 361003, PR China.*

*Corresponding author: Qin Lin, M.D., Ph.D.
Tel: 86-592-2137201 Fax: 86-592-2137189 E-mail:*[*linqin05@163.com*](mailto:linqin05@163.com)

**The information of the patient with SCC:**

A postoperative case with multifocal SCC, male, 77 years old.

**Actual treatment plan:**

The patient was actually irradiated with 25×2 Gy (50 Gy) to CTV and 37×2 Gy (74 Gy) to GTV. The CTV included the entire scalp.

**
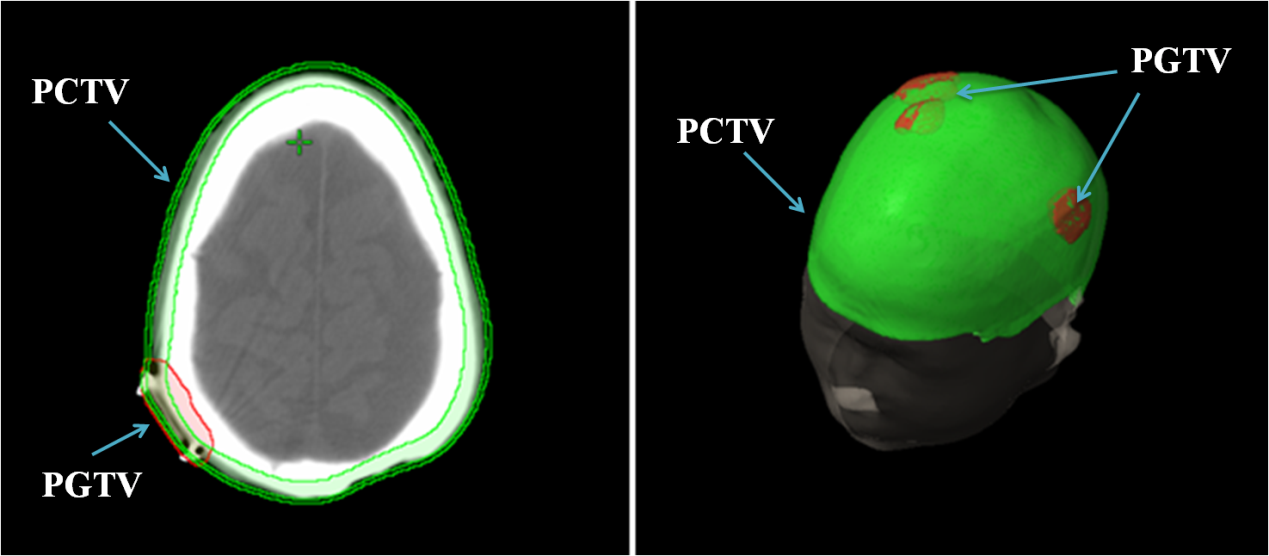
**

**Figure S1.** The PCTV and PGTV for the patient with SCC.

**
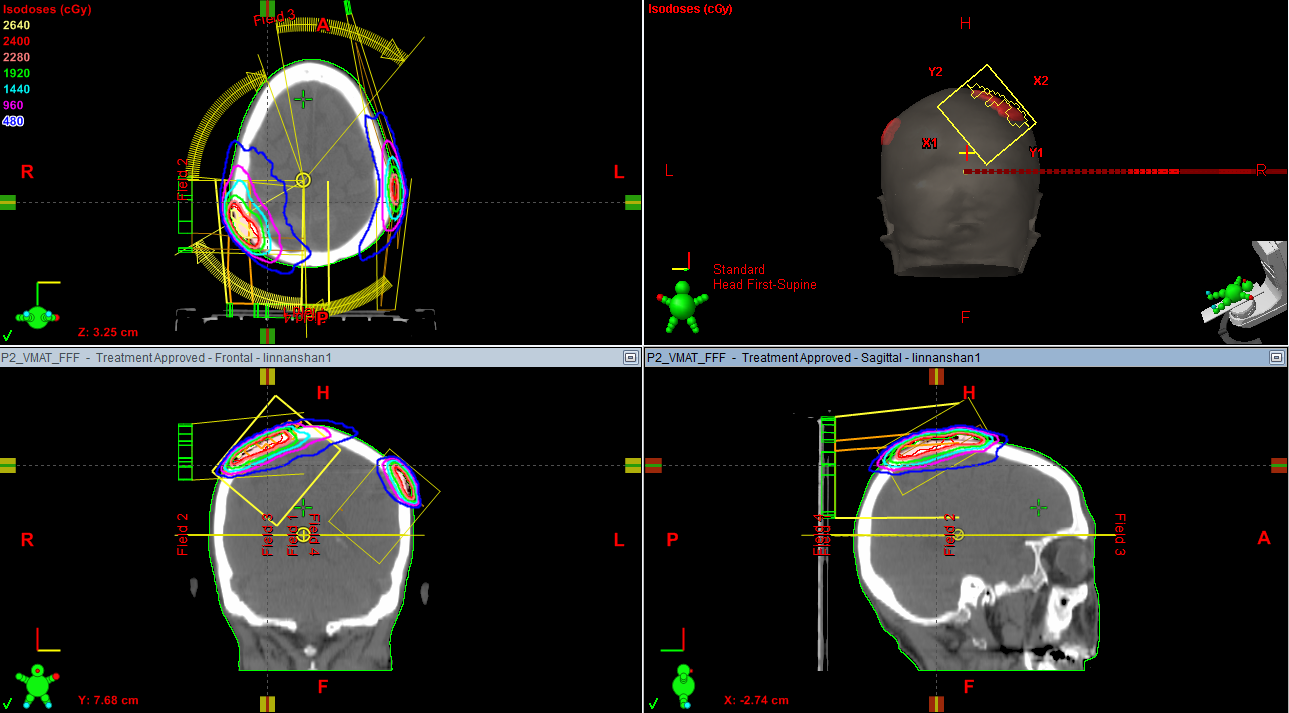
**

**Figure S2.** The beam setup and isodose distributions for treatment plan of second phase. Prescribed dose was 12×2 Gy (24 Gy) to PGTV. For the treatment plan of second phase, the patient with SCC was irradiated with 12×2 Gy (24 Gy) to GTV to boost the GTV up to 74Gy. Four double partial arcs with a maximum individual length of 60° were adopted in this plan.

**
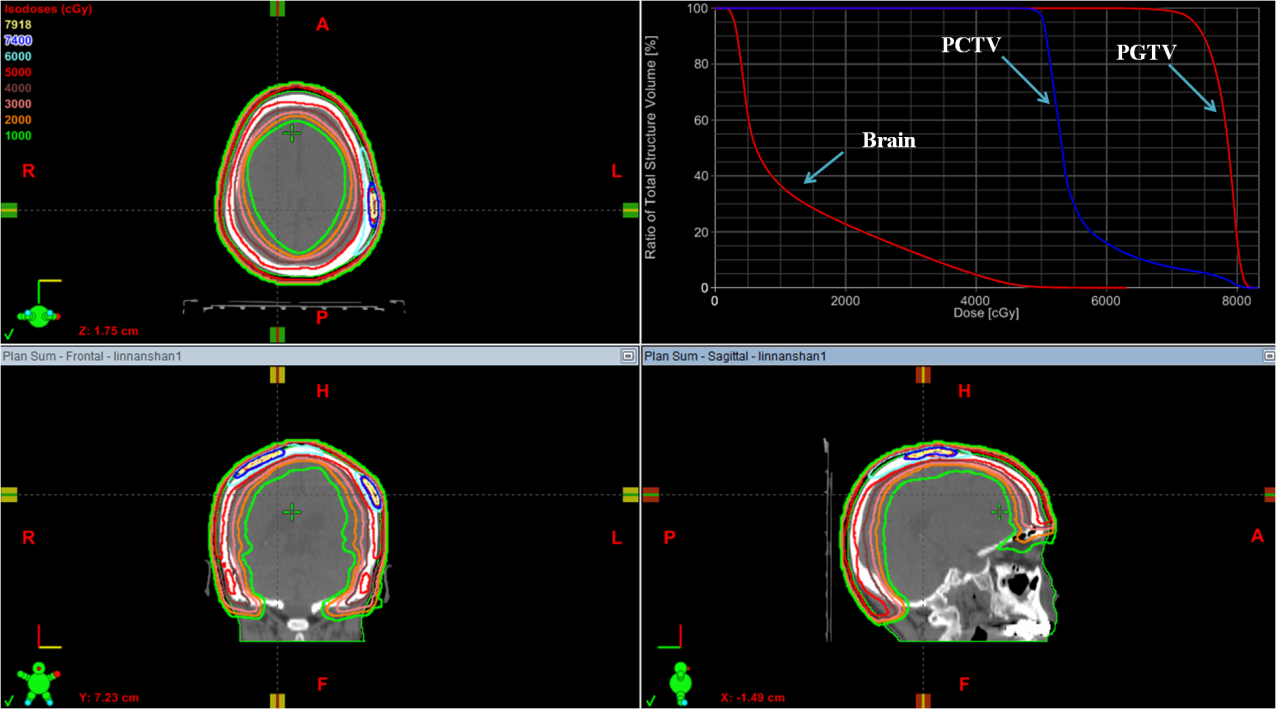
**

**Figure S3.** The isodose distributions and hose-volume histogram (DVH) for the sum plan with 25×2 Gy (50 Gy) to PCTV and 37×2 Gy (74 Gy) to PGTV.

**File S1.** The data (file data_PONE-D-14-34378R1.docx) underlying the findings in present study.
